# Antioxidant activity and comparative RNA‐seq analysis support mitigating effects of an algae‐based biostimulant on drought stress in tomato plants

**DOI:** 10.1111/ppl.70007

**Published:** 2024-12-20

**Authors:** Paolo Cerruti, Cristina Campobenedetto, Elisa Montrucchio, Chiara Agliassa, Valeria Contartese, Alberto Acquadro, Cinzia Margherita Bertea

**Affiliations:** ^1^ Department of Agricultural, Forest and Food Sciences University of Turin Grugliasco Italy; ^2^ Green Has Italia Canale Italy; ^3^ Plant Physiology Unit, Department of Life Sciences and Systems Biology University of Turin Torino Italy; ^4^ Present address: AIT‐ Austrian Institute of Technology GMBH Vienna Austria

## Abstract

Drought is a significant global environmental stress. Biostimulants offer a sustainable solution to enhance crop tolerance and mitigate productivity losses. This study assessed the impact of foliar application of ERANTHIS®, a biostimulant derived from the algae *Ascophyllum nodosum* and *Laminaria digitata* and yeast extracts, on tomato plants under mild water stress. Evaluations were conducted at 5 and 24 hours after the third treatment. Under optimal water conditions, the biostimulant showed a priming effect, with an early increase of stress markers and a timing‐specific modulation of ROS non enzymatic and enzymatic ROS scavenging activities. Under drought stress, the biostimulant later decreased stress markers, by aligning the majority of analyzed ROS scavengers closer to levels in well‐irrigated plants. Transcriptome analysis using RNA‐seq data revealed differentially expressed genes (DEGs) and multivariate data highlighted groups of co‐regulated genes (k‐means clustering). Genes involved in water channel activity, transcription regulator activity, and oxidoreductase activity were significantly modulated. Cluster analysis identified distinct gene clusters influenced by the biostimulant under optimal conditions, including early responses (cell wall modification, hormone signaling) and late responses (RNA modification, nutrient uptake process). Under water stress, early responses involved actin filament organization and MAPK signaling, while late responses were related to plasma membrane components and cell wall organization. This study, integrating biochemical and transcriptomic data, provides a comprehensive understanding of how a biostimulant primes plants under optimal conditions and mitigates water stress effects, offering valuable insights for sustainable agriculture.

## 1 INTRODUCTION

Although the demand for food is ever greater, climate changes make the productive capacity of crops increasingly limited (Ahluwalia et al., 2021). Indeed, more than 50% of global yield losses of economically important crops are related to abiotic stress factors (Junaid & Gokce, 2024). Amongst environmental stressors, drought stress, is one of the worldwide most widespread stressor and it leads to losses, both in terms of quantity of production and crop quality (Campobenedetto et al., 2021; Sun et al., 2020). According to the United Nations Convention to Combat Desertification (UNCCD), water shortage caused global economic losses of roughly USD 124 billion from 1998 to 2017 (https://www.unccd.int). Water shortage damages plants and limits their growth by modifying their morphological, physiological and biochemical responses (Seleiman et al., 2021). Over the years, various remedies have been used to deal with losses in productivity due to water shortages, such as the intensive use of chemical fertilizers. These have been proven to be useful for the plant in many cases, but harmful to the environment (Liliane & Charles, 2020). For this reason, it became necessary to find solutions capable of improving production and quality performance, without damaging the environment.

Among these, biostimulants represent a concrete and effective help (Mandal et al., 2023) and their market has been spreading worldwide. Indeed, the global biostimulant market, valued at approximately US$2.9 billion in 2022, is expected to reach nearly US$4.9 billion by the end of 2030. Moreover, the market is forecasted to experience a robust CAGR of 7.8% from 2023 to 2030 (https://www.fairfieldmarketresearch.com). Biostimulants enhance plant growth, productivity, and stress resilience through the synergistic effects of bioactive compounds by acting at very low dosages (Mannino et al., 2020). These innovative products derive from various natural sources, including plant extracts, seaweed, and microbial derivatives, offering a promising sustainable alternative to traditional chemical treatments. The complexity of their formulation makes it difficult to understand their mode of action, even though many researchers report their agronomic effectiveness in adverse growth conditions (Yakhin et al., 2017).

Tomatoes are currently ranked as the seventh most extensively cultivated crop worldwide, with production overtaking 186 million tons as reported in 2022 (FAO, 2022). However, tomato production is severely affected by drought stress (Liang et al., 2020; Pervez et al., 2009). Tomato (*Solanum lycopersicum* L., chromosome number, 2n = 24) is indigenous to regions including Central America, South America, and the southern regions of North America. This diploid plant has seen a surge in interest in the Micro Tom cultivar as a model organism, especially following the complete sequencing of its genome (Kobayashi et al., 2014). The Micro Tom cultivar, developed for ornamental purposes through the crossbreeding of the Florida Basket and Ohio 4013–3 cultivars, is prized for its advantageous traits such as a short life cycle, self‐fertility, high homozygosity, controlled pollination and hybridization, seed production capability, a compact genome size of 950 Mb, and the feasibility of asexual reproduction via grafting (Kobayashi et al., 2014). Therefore, studies on the Micro Tom cultivar could help in fastening the understanding of biostimulant mode of action on tomato.

This study aims to validate the effectiveness of ERANTHIS®, a biostimulant composed of brown seaweed extracts (*Laminaria digitata* and *Ascophyllum nodosum*) and yeast extracts, in promoting drought mitigation in the Micro Tom cultivar. The research seeks to contribute to the development of sustainable and effective systems to address drought‐related climate challenges, by considering their comprehensive mode of action. To this purpose, we analyzed specific biochemical and transcriptomic responses induced by the biostimulant in tomato leaves under well‐watered and stress conditions by performing spectrophotometric assays on oxidative stress markers and ROS scavengers and RNA‐seq analyses.

## 2 MATERIALS AND METHODS

### 2.1 Plant material, biostimulant and experimental conditions

*Solanum lycopersicum* (tomato) seeds, var. Micro Tom, were purchased by Pan America Seed (Chicago). The seeds were sown in rock wool cubes and then transferred in pots containing 50% of unfertilized peat and 50% of expanded clay. The trial was conducted in a greenhouse, at 17–30°C under sunlight with LED lamps in order to maintain 100 μmol m^−2^ s^−1^ during the light period, in a 16/8 h diurnal cycle with 60% relative humidity.

**FIGURE 1:**
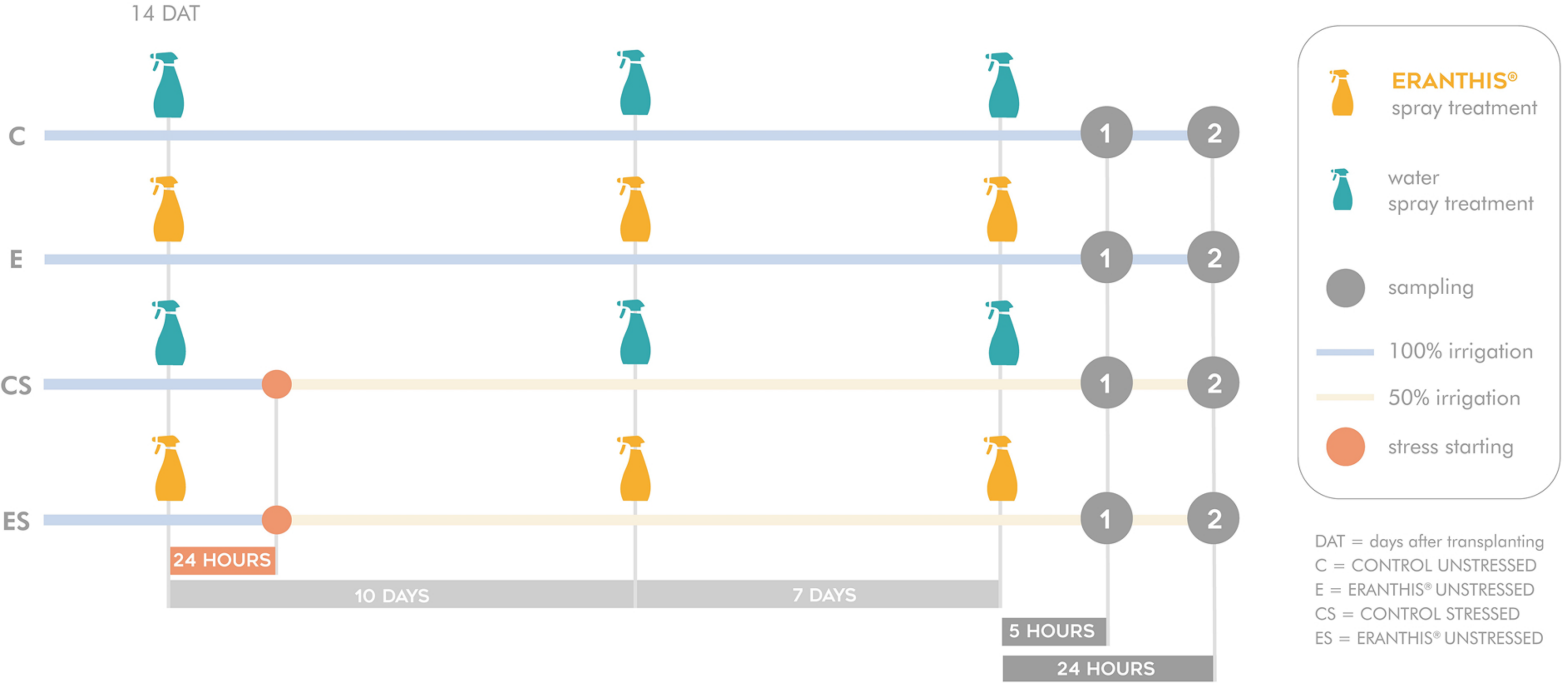
Schematic representation of the experimental design. At the beginning, all plants were irrigated with full water supply. At 14 days after transplanting (DAT), the first biostimulant treatment was performed on E and ES plant groups, while control plants (C and CS) were water sprayed. At 24 hours after the first biostimulant treatment, water stress was induced in half of the plants (CS and ES) by reducing the water intake by 50%, resulting in 30% less water at the end of the trial. The other half continued to be well‐irrigated (C and E). Plants were then water sprayed (C and CS) or biostimulant treated (E and ES) twice (at 10 days and 17 days after the first biostimulant treatment) before leaf sampling. Plant material was collected at 5 (timing 1) and 24 (timing 2) hours after the third biostimulant treatment.

A total of 112 plants were used, divided into four different groups: 1. Control unstressed (C), 2. ERANTHIS® unstressed (E), 3. Control stressed (CS) and 4. ERANTHIS® stressed (ES) (Figure 1). Therefore, the trial was bifactorial: C together with CS groups and E together with ES groups defined the treatment factor (control versus ERANTHIS® treated plants), whereas C together with E groups and CS together with ES groups defined the irrigation factor (unstressed versus mild water stressed plants). The study was focused on biochemical aspects like antioxidant enzyme activities, H_2_O_2_, osmolyte and non‐enzymatic antioxidant content, and molecular RNA‐seq analysis. The plants were treated three times by foliar spray with the biostimulant at a dose of 2 mL l^−1^ (E and ES), or tap water (C and CS). The three spray treatments occurred at the fourth true leaf appearance at 14 days after transplanting (DAT), at the beginning of flowering (24 DAT) and at seven days after the second treatment. At 24 hours after the first application which was the priming treatment, water stress was induced in group 3 (CS) and 4 (ES) by reducing irrigations by 50%. Stress continued until the end of the experiment. Therefore, by considering water supply from transplanting to the end of the trial, stressed plants received 30% less water with respect to unstressed plants.

Leaf samples were collected at two time points: the first sampling (timing 1) occurred at 5 hours after the third treatment in the early afternoon at 3 p.m., while the second sampling (timing 2) took place at 24 hours after the third treatment, the following morning at 10 a.m. Three biological replicates were done per group, each one composed of three plants.

The collected plant material was stored in a − 80°C freezer before performing biochemical and RNA‐seq analyses.

ERANTHIS® was provided by Green Has Italia S.p.A. The product obtained the conformity as biostimulant for drought stress in 2023 in accordance with Reg. EU 2019/1009. Its formulation contains brown algae extracts from *Laminaria digitata* and *Ascophyllum nodosum*, yeast extracts and selected peptidic‐sources. Its pH (in 1% w/w water solution) is of 5.0 ± 0.5 (measured on three different replicates and expressed as mean ± standard deviation) and its electric conductivity is of 250 μS cm^−1^. Its chemical characterization revealed the presence of bioactive phenolic compounds, probably correlated to the high antioxidant capacity of the formulation (Campobenedetto et al., 2021).

### 2.2 Biochemical analysis on leaves

Leaf samples collected at 5 and 24 hours after the third biostimulant treatment were used for both biochemical and transcriptomic analyses. Three biological replicates, each one composed of three plants for each group, were employed for all the analyzed parameters.

#### 2.2.1 Hydrogen Peroxide (H_2_O_2_ ) extraction and quantification

The hydrogen peroxide content was detected according to Velikova and colleagues (Velikova et al., 2000). Powdered fresh leaves (0.3 g) were homogenized in 0.1% (w/v) Trichloroacetic Acid (TCA). Samples were mixed by vortexing, centrifuged at 12 000 *g* for 15 min and then incubated at 4°C for about three hours. Then, the supernatant was used and 10 mM Potassium Phosphate buffer (pH 7.0) and 1 M Potassium Iodide (KI) were added to reach 1 mL of final volume. The absorbance was read at 390 nm, after an incubation of 20 min at room temperature. The H_2_O_2_ content was calculated based on a standard curve.

#### 2.2.2 Antioxidant enzyme extraction and activity

Antioxidant enzymes and total soluble proteins were extracted according to (Contartese et al., 2015). The resulted supernatant was used for enzymatic assays following the evaluation of soluble protein content in accordance with the method of Bradford (1976). All enzyme activity data were expressed as specific activity on mg of total proteins.

Superoxide dismutase (SOD, EC 1.15.1.1)‐ SOD activity was determined by estimating one unit of SOD as the amount of protein per milligram causing 50% inhibition of Nitro Blue Tetrazolium (NBT) reduction. The reaction mix consisted of 1 mL final volume containing 50 mM Sodium Phosphate buffer (pH 7.8), 13 mM Methionine, 0.1 mM EDTA, 75 μM NBT, 2 μM Riboflavin and 50 μL of enzyme extract. The reaction was developed under a light source for 15 min. A positive control without enzyme extract was placed under the light, to completely develop the reaction, and a negative one containing the enzyme extract placed in the dark, to avoid the reaction and used to reset the instrument. The absorbance was detected at 560 nm (Campobenedetto et al., 2020).

Catalase (CAT, EC 1.11.1.6)– CAT activity was measured spectrophotometrically by monitoring the decrease of hydrogen peroxide absorption (ɛ = 39.4 mM^−1^ cm^−1^) at 240 nm for 120 sec. Briefly, 1 mL reaction mixture was prepared, containing 50 mM Sodium Phosphate buffer (pH 7.0) and 50 μL of enzyme extract and initiated by the addition of 15 mM H_2_O_2_. The activity was measured by monitoring the μmol of decomposed H_2_O_2_ per minute (Campobenedetto et al., 2020).

Peroxidase (POX, EC 1.11.1.7) – POX activity was measured spectrophotometrically by following the oxidation of Guaiacol (Ɛ = 26.6 mM^−1^ cm^−1^) in the presence of hydrogen peroxide. 100 μL of enzyme extract was added to 50 mM Sodium Phosphate buffer (pH 7.0), 0.27 mM H_2_O_2_ and 0.33 mM Guaiacol in 1 mL of final volume. The reaction started with guaiacol, was left to develop in the dark for 15 minutes and the absorbance increase read at 470 nm (Campobenedetto et al., 2021).

GST (EC 2.5.1.18)‐ Glutathione‐S‐Transferase activity was evaluated by monitoring the increase in absorbance at 340 nm due to the conjugation of 1‐Chloro‐2,4‐Dinitro‐Benzene (CDNB) to reduced Glutathione (GSH) for 15 min. 1 mL of the reaction solution contained 100 mM Potassium Phosphate buffer (pH 7.0; KH_2_PO_4_/K_2_HPO_4_), 1 mM GSH, 1 mM CDNB and 50 μL of enzyme extract. The reaction was started by adding CDNB (Campobenedetto et al., 2020).

#### 2.2.3 Non enzymatic antioxidant and osmolytes

Leaf non‐ enzymatic antioxidants were extracted and quantified by using specific protocols. Non‐protein thiol content was measured by using the same protein extracts used to evaluate antioxidant enzyme activities.

Proline (PRO)‐ Proline was extracted according to Carillo and Gibon (Carillo & Gibon, 2011). The powdered fresh leaves (50 mg) were homogenized in Ethanol 70% (v/v) and incubated for two days at 4°C. 1 mL of reaction mix, containing 1% (w/v) Ninhydrin dissolved in 60% (v/v) Acetic acid and 20% (v/v) Ethanol, was added to the obtained supernatant. The mixture was heated at 95°C for 20 min and then centrifuged at 10 000 *g* for 1 min. The absorbance was read at 520 nm and the Proline content was calculated based on a standard curve.

Glycine betaine (GB)‐ To extract glycine betaine, the powdered fresh leaves (5–10 mg) were homogenized in 1.5 mL of 2 N Sulfuric acid, heated at 60°C for 10 min and centrifuged at 14 000 *g* for 10 min at room temperature. Then, 125 μL of supernatant was mixed with 50 μL of KI. Samples were incubated at 4°C for 16 hours to let the formation of glycine betaine crystals occur. After a centrifugation at 14 000 *g* for 30 minutes at 0°C and the elimination of supernatant, the pellet (crystals) was dissolved in dichloroethane. Two hours later, the absorbance was read at 365 nm. The glycine betaine content was calculated based on a standard curve (Valadez‐Bustos et al., 2016).

Non‐protein thiols (NPSH)‐ Non‐protein thiol content was evaluated by measuring the formation of the free glutathione. After adding protein extracts to 25% (w/v) TCA, the mixture was centrifuged at 12 000 *g* for 20 min at 4°C and supernatants were added to 0.6 mM 5,5′‐dithiobis‐2‐nitrobenzoic acid (DTNB; Ellman's reagent, ɛ = 14.150 mM^−1^ cm^−1^) and dissolved in 0.1 mM Sodium Phosphate buffer (pH 8.0). The absorbance was measured at 412 nm (Campobenedetto et al., 2020).

### 2.3 Statistical analysis

Data are expressed as mean values ± standard deviation (SD) of three different biological replicates. All statistical analyses were performed by using the IBM® SPSS Statistics 28.0 software. Significant differences were evaluated by performing a two‐way ANOVA followed by Tukey's post‐hoc test (*p*‐value ≤0.05). The two independent variables were the irrigation factor (unstressed or stressed), and the treatment factor (control or ERANTHIS®‐treated). In the presence of independent variable interaction, data were analyzed with a *t*‐test (*p* < 0.05) by comparing unstressed plant (C vs. E) and stressed plant groups (CS vs. ES) separately. Instead, if no interaction occurred, a *t*‐test (*p* < 0.05) was performed by comparing control plant groups (C and CS) against ERANTHIS®‐ treated plant groups (E and ES).

### 2.4 RNA‐ seq

#### 2.4.1 Total RNA Isolation

Total RNA was isolated from leaves using TRIzol® reagent, with modifications for enhanced yield as described in (Wang et al., 2012). The total RNA was further purified using the RNeasy® Mini Kit (Qiagen) following the RNA clean‐up protocol. Total RNA concentration was determined by spectrophotometry (Ultrospec 3000, Amersham Pharmacia Biotech), and quality assessment was carried out with the RNA 6000 Nano kit using an Agilent 2100 Bioanalyzer (Agilent Technologies), following the manufacturer's guidelines. RNA was isolated from control and ERANTHIS®‐treated leaves, both under optimal and stress conditions, sampled at 5 hours and 24 hours post‐treatment, each with three biological replicates, resulting in 24 samples.

#### 2.4.2 RNA‐Seq Analysis

Libraries were constructed using the TruSeq Stranded Total RNA LT Sample Prep Kit for Illumina® (NEB) at Macrogen Europe and index codes were added to attribute sequences to each sample. Libraries were sequenced on an Illumina platform and 150 bp paired‐end reads were generated. Raw sequencing data underwent quality control and adapter trimming using the fastp tool (https://github.com/OpenGene/fastp). The reference genome (SL3.0; GCF_000188115.4_SL3.0_ genomic.fna) was indexed, and the processed reads were aligned using the bowtie2 aligner (http://bowtie-bio.sourceforge.net/bowtie2/index.shtml). Alignment files were generated for each replicate and counts of aligned reads were calculated utilizing samtools (http://www.htslib.org/). The read counts were the basis for differential gene expression analysis.

### 2.5 Identification of Differentially Expressed Genes (DEG)

The identification of DEGs was conducted using the DESeq2 (https://bioconductor.org/packages/release/bioc/html/DESeq2.html). Normalized count data served as input for pairwise comparisons among three biological replicates across distinct conditions (ERANTHIS® vs. Control). Comparisons focused on those reflecting the biostimulant effects were prioritized, excluding the ones solely indicative of water stress (e.g., Control stressed vs. Control unstressed). Specifically, pairwise comparisons were as follows: ERANTHIS® unstressed versus Control unstressed (E vs. C) and ERANTHIS® stressed versus Control stressed (ES vs. CS), at two time points post‐treatment (5 hours and 24 hours), yielding the following contrasts: (1) E vs. C at 5 hours, (2) E vs. C at 24 hours, (3) ES vs. CS at 5 hours, and (4) ES vs. CS at 24 hours. This experimental design resulted in 24 sequencing libraries encompassing all conditions and time points.

Differentially expressed genes (DEGs) identified by DESeq2 were visualized using Venn diagrams (https://bioinformatics.psb.ugent.be/webtools/Venn/), delineating the common and unique DEGs across the four experimental comparisons. For the DEGs, functional enrichment analysis was performed using the ShinyGO platform (http://bioinformatics.sdstate.edu/go). This analysis used a False Discovery Rate (FDR) threshold set to FDR ≤0.1, limiting false positives to less than 10%. Pathways were annotated employing integrated databases in ShinyGO, including KEGG for metabolic pathways, as well as GO classifications for Cellular Component (CC), Biological Process (BP), and Molecular Function (MF).

### 2.6 Multivariate data analysis

The Genesis software suite (http://genome.tugraz.at) facilitated clustering, analysis, and visualization tasks. All the data points were expressed in log_2_FC, and specifically normalized. In particular, in both experiments (with and without water deprivation), all data collected at 5 hours were normalized with the control condition at 5 hours (C5), while for data collected at 24 hours, the control condition at 24 hours (C24) was used as reference for the normalization. Gene expression data were subjected to unsupervised clustering using the k‐means algorithm, with the Euclidean distance serving as the similarity metric. To optimize the number of clusters (k), we varied k from 1 to 100, employing the figure of merit (FOM) to assess predictive power and guide selection. Cluster robustness was also confirmed via repeated runs with different initial conditions. The chosen k value corresponds to the lowest FOM, indicative of the most accurate and stable clustering outcome. Post‐analysis involved manual selection of pertinent clusters, for detailed exploration with ShinyGO (set as explained in the previous paragraph) in which genes from clusters with similar trends were merged to do a common analysis. Finally, where possible, association networks were constructed to observe interactions between clustered genes sharing the same trend with STRING (https://string-db.org/, minimum required interaction score: 0.5).

## 3 RESULTS AND DISCUSSION

Biostimulants are products increasingly used in agricultural systems, thanks to their potential and characteristics. They normally contain substances of natural origin, often deriving from industrial waste, fitting into a circular economy perspective. ERANTHIS®, the biostimulant tested in this study, is based on the seaweeds *Ascophyllum nodosum* and *Laminaria digitata* and yeast extracts. Both these components are already known to have biostimulant activity (Van Oosten et al., 2017). Seaweed extracts contain complex polysaccharides, fatty acids, vitamins, phenolic compounds, and are useful for different purposes, like antioxidant activity (Battacharyya et al., 2015). Yeast extracts are known to be effective in different mechanisms, like carbohydrate accumulation, cell division and enlargement stimulation, protein and nucleic acid synthesis, and chlorophyll formation (Mohamed et al., 2021). Thanks to their formulation, biostimulants are known to be involved in mitigating different kinds of abiotic stress (du Jardin, 2015). The role of *Ascophyllum nodosum* as anti‐stress factor was already observed on crops subjected to salinity (Attia et al., 2023) or drought stress (do Rosário Rosa et al., 2021), while the effect of *Laminaria digitata* and yeast extract is less studied, even if their use as stress mitigators is reported in several cases (Mannino et al., 2020).

In this study, the biostimulant ERANTHIS® was tested on tomato plants under optimal and drought stress conditions, in order to deepen the understanding of its anti‐stress mode of action. This last aspect is supported by the product formulation and by its chemical characteristics highlighted in a previous work published by Campobenedetto et al., (2021). Indeed, as demonstrated through spectrophotometric analysis, ERANTHIS® is a rich source of phenolic compounds, mainly belonging to the flavonoid and flavanol families. These molecules are probably derived from seaweed extracts of *Ascophyllum nodosum* and *Laminaria digitata*, that are rich in antioxidant compounds (Corsetto et al., 2020). The product anti‐stress mode of action was evaluated at 5 and 24 hours after the third treatment by monitoring leaf oxidative status and the antioxidant machinery and by performing an RNA‐seq analysis. The two analyzed timings allowed us to discriminate between early (5 hours) and late (24 hours) responses to the ERANTHIS® third treatment. These time sampling points were chosen based on our previous physiological and agronomical findings (data not shown). This comprehensive approach allowed us to shed some light on the mode of action of ERANTHIS® also by discriminating its activity on tomato leaves under both optimal and water stress conditions.

### 3.1 Biochemical oxidative stress markers

**FIGURE 2:**
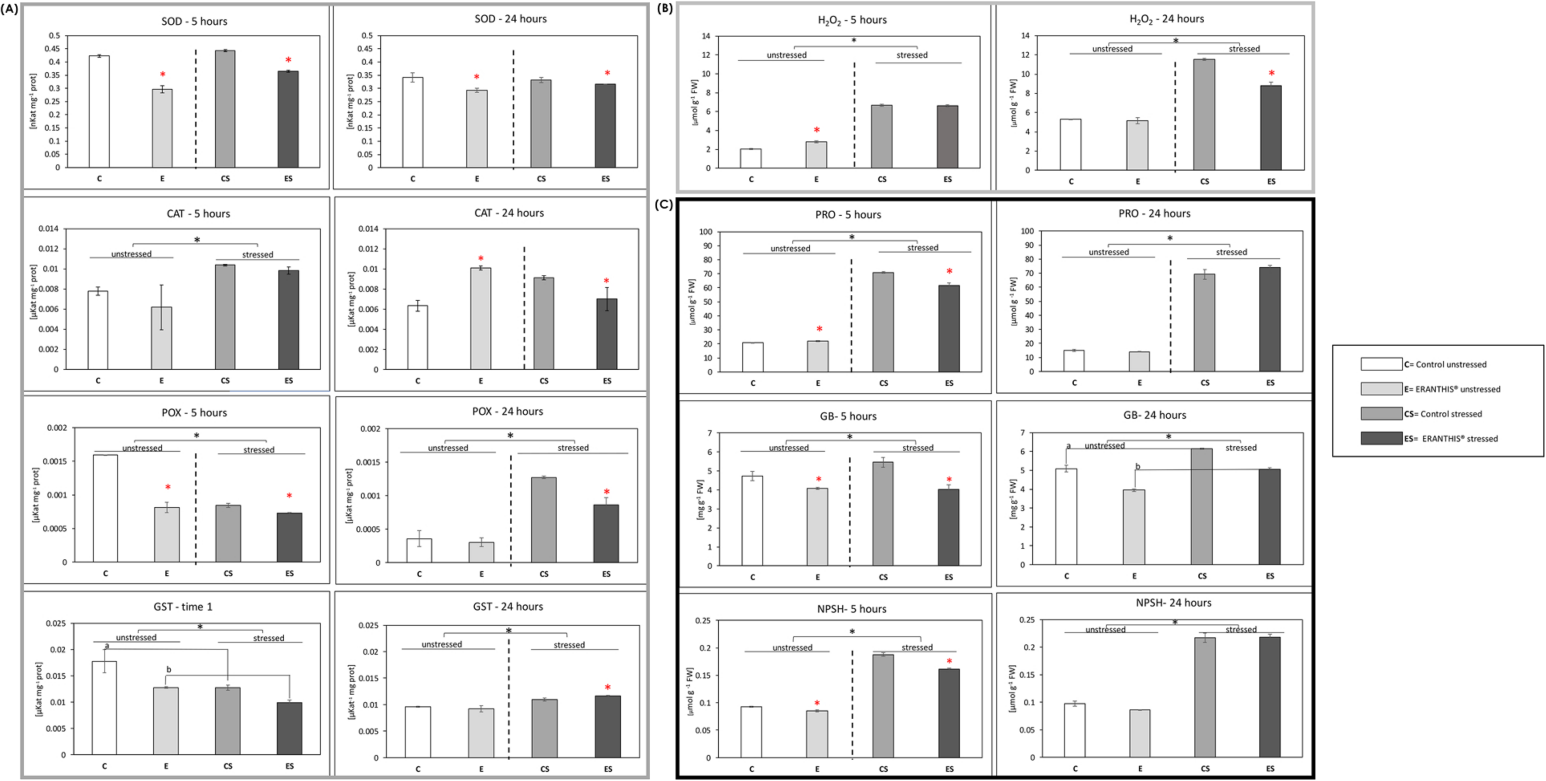
Biochemical analysis on leaves. The dark grey panel (A) refers to the antioxidant enzyme activities; the light grey panel (B) refers to hydrogen peroxide content; the black panel (C) refers to the non‐enzymatic antioxidant and osmolyte levels. Panel (A) includes Superoxide Dismutase, SOD, Catalase, CAT, Peroxidase, POX, Glutathione‐S‐Transferase, GST activities. Panel (C) includes proline, PRO, glycine betaine, GB, non‐protein thiols, NPSH levels. For each panel, the left column refers to data collected at 5 hours after treatment, while the right column represents data collected at 24 hours after treatment. Three biological replicates, each composed of three plants for each group, were analyzed. Two‐way ANOVA followed by Tukey's test (*p* ≤ 0.05) was performed. The black asterisk (*) indicates the statistical difference between unstressed and stressed plants (irrigation factor). When the interaction between the condition of irrigation and treatment occurred (black vertical dotted line), the results were analyzed separately (C vs. E, CS vs. ES) by applying a t‐test (*p* < 0.05, red asterisk). When the interaction between the condition of irrigation and treatment did not occur, untreated plants (C and CS) were compared to treated ones (E and ES) by applying the t‐test (p < 0.05). The two groups were denoted by different letters “a” and “b” when significantly different.

To evaluate the oxidative status of the plant under optimal and drought stress conditions, the content of hydrogen peroxide, non‐enzymatic ROS scavengers like proline, glycine betaine and non‐protein thiols and enzymatic ROS scavengers like SOD, CAT, POX and GST was evaluated on tomato leaves. In particular, H_2_O_2_ is a ROS species involved in different mechanisms like stress perception (Cheeseman, 2007). It is produced at a basal level during the plant's life and increased by oxidative stress, thus representing a good candidate as an oxidative stress marker. Regarding the antioxidant machinery, superoxide dismutase, catalase, peroxidase and glutathione‐S‐transferase are ROS scavenger enzymes involved in the oxidative stress response mechanism. In detail, superoxide dismutase leads to H_2_O_2_ and O_2_ formation from two superoxide anions, acting in the first line of defense against ROS activity (Liochev & Fridovich, 2007). Catalase catalyzes the dismutation of H_2_O_2_ into H_2_O and O_2_ (Feierabend, 2005). Peroxidase transforms the hydrogen peroxide into water and molecular oxygen, while GST catalyzes the formation of glutathione S‐conjugates between glutathione and xenobiotics (Quan et al., 2008; Cooper & Hanigan, 2018). Among non‐enzymatic antioxidants, thiols are non‐protein molecules that maintain the integrity of the photosynthetic membranes under oxidative stress (Salbitani et al., 2023). Glycine betaine is strictly related to the stress response, acting at a cellular level with different mechanisms, such as cell osmotic pressure and ROS detoxification (Majumder et al., 2010). In the end, proline is an osmolyte involved in the maintenance of the cell osmotic balance (Hayat et al., 2012). All biochemical data collected at 5 and 24 hours after the third biostimulant treatment were resumed in Figure 2.

#### 3.1.1 ERANTHIS® changes leaf oxidative status in optimal conditions

Although biostimulants are known to be more effective under stress conditions (Van Oosten et al., 2017), ERANTHIS® effect on the tomato leaf oxidative status was evaluated also under well‐irrigated conditions. Our data suggested that the product seems indeed to change tomato oxidative leaf status even under optimal conditions.

At 5 hours post‐treatment, the content of H_2_O_2_ was slightly increased (Figure 2B; H_2_O_2_–5 hours) by the treatment. Interestingly, Kambona and colleagues showed that a first mild stress condition can lead to the increase of stress signal molecules, like H_2_O_2_, that can make the plant more responsive to a second stress event (Kambona et al., 2023). At this timing (5 hours), the activity of SOD, POX and GST were decreased by the product application, whereas CAT activity did not show any significant change (Figure 2(A), 5 hours).

Considering the non‐ enzymatic antioxidants, the treatment led to an early increase of PRO content and a decrease of GB and NPSH at 5 hours (Figure 2C; 5 hours). The proline increase observed at this time could be due to a mild stress‐mimicking effect induced by the product, as highlighted for H_2_O_2_. Hosseinifard and colleagues observed that the application of exogenous proline can induce a priming effect making the plant able to respond stronger and faster when exposed to abiotic stress (Hosseinifard et al., 2022). Moreover, the priming performed with proline can also increase the activity of some ROS scavenger enzymes, such as CAT, as observed by Shafiq et al., (2018).

Interestingly, at 24 hours post‐treatment, treated plants showed increased CAT and decreased SOD activity, whereas no difference was observed in POX and GST activity (Figure 2A; 24 hours). In particular, the strongly increased activity of CAT could be induced by the rise in H_2_O_2_ levels observed at 5 hours after the third treatment. As a possible consequence, H_2_O_2_ values measured on plants treated with ERANTHIS® were similar to the untreated control at 24 hours (Figure 2B; 24 hours), despite the reduced SOD activity (Anderson, 2002). Similar results were obtained by Agliassa and colleagues who observed an increase in CAT activity in pepper leaves treated with a biostimulant under optimal conditions (Agliassa et al., 2021).

Considering the non‐ enzymatic antioxidants, glycine betaine was the only variable significantly changed at 24 hours after the treatment (Figure 2C; GB‐ 24 hours). The glycine betaine reduced level is consistent with the previous timing (5 hours), thus suggesting a potential regulation exerted by ERANTHIS® on osmolyte content.

#### 3.1.2 ERANTHIS® mitigates oxidative response in stress conditions

Under stress conditions, ERANTHIS® treated plants showed a different response with respect to untreated plants. In general, oxidative stress marker values of treated leaves were closer to well‐irrigated plants than untreated stressed plants (Figure 2).

At 5 hours post‐treatment, the presence of stress significantly influences all biochemical variables, with the exception of SOD (Figure 2, 5 hours). However, SOD reduction in ERANTHIS® treated plants was consistent under both optimal and drought stress conditions (Figure 2A; SOD‐ 5 hours). Similarly, Gil‐Ortiz and colleagues observed the decrease of SOD activity following biostimulant application in plants grown under drought stress (Gil‐Ortiz et al., 2023).

At the same sampling time (5 hours), under stress conditions, the biostimulant application differently regulated all the biochemical variables, with the exception of H_2_O_2_ level (Figure 2B; 5 hours) and CAT activity (Figure 2A; 5 hours). The increased content of H_2_O_2_ and CAT in drought stress conditions was already reported in the literature for different crop species, like tomato, maize, wheat, and rice (Z. Zhang et al., 2019).

At this time point of 5 hours, POX activity was reduced by drought stress and eventually reduced by ERANTHIS® application (Figure 2A; POX‐ 5 hours). Moreover, drought stress led to a decrease of GST activity and therefore to an increased thiol content (Zagorchev et al., 2013), as we observed at 5 hours after the third biostimulant treatment (Figure 2A; GST‐ 5 hours, Figure 2C; NPSH‐ 5 hours). The difference between stressed and unstressed plants observed in this work agrees with other studies in which in different species, a higher level of thiols was measured when plants were grown in stress conditions (Koramutla et al., 2021; L. Zhang et al., 2021). ERANTHIS® application influenced NPSH content and GST activity (two‐way ANOVA, no interaction with stress). These parameters are partially correlated, glutathione being the main non‐protein thiol in plants (Noctor et al., 2012). Tomato plants, when treated with ERANTHIS®, showed a stronger GST activity reduction and a significant decrease of thiols with respect to untreated stressed plants. Therefore, treated stressed plants showed values closer to those of plants grown under optimal conditions. In the same way, Ozfidan‐Konakci and colleagues demonstrated the mitigation effect of a biostimulant based on humic acids, able to enhance the tolerance to cadmium stress and decrease GSH levels in treated wheat plants (Ozfidan‐Konakci et al., 2018).

ERANTHIS® treatment influenced PRO and GB accumulation in stress conditions at 5 hours after the treatment. In particular, the observed glycine betaine increase induced by drought stress (Figure 2C; GB‐ 5 hours) is consistent with several studies reported in the literature (Annunziata et al., 2019; Sohag et al., 2020). ERANTHIS® treatment led to a decrease of GB with respect to untreated stressed plants at both time points (Figure 2C; GB). Similarly, drought stress led to a significant increase of PRO at both time points with respect to control plants (Figure 2C; PRO), but the treatment induced a significant decrease of proline content at 5 hours. Francesca and colleagues showed that in tomato plants grown under drought stress and treated with a protein hydrolysate based biostimulant, the proline content was lower compared to untreated plants (Francesca et al., 2022).

At 24 hours post‐treatment, the presence of stress significantly influenced all the analyzed biochemical variables, with the exception of SOD and CAT (Figure 2, 24 hours). However, SOD reduction in ERANTHIS® treated plants was still consistent under both optimal and drought stress conditions (Figure 2A; SOD‐ 24 hours). Moreover, ERANTHIS treatment reduced CAT activity in stressed plants (Figure 2A; CAT‐24 hours). Similarly, Macias‐Benitez and colleagues found out that a biostimulant with high antioxidant capacity was able to help pepper plants to tolerate oxidative stress, caused by ozone treatment, by decreasing all the assayed antioxidant enzymatic activities, including CAT (Macias‐Benitez et al., 2021).

At the sampling time of 24 hours, the biostimulant application differently regulated the majority of the biochemical variables under stress conditions. An exception was observed for PRO and NPSH which levels were increased by the treatment (Figure 2C; 24 hours). In particular, the biostimulant application differently regulated POX and GST activity (Figure 2A; 24 hours) together with H_2_O_2_ levels in tomato leaves (Figure 2B; 24 hours). In our experimental conditions, drought stress led to a significant increase of these parameters with respect to untreated control plants. When ERANTHIS® was applied, tomato leaves showed a lower increase in H_2_O_2_ content (Figure 2B; 24 hours), as well as POX activity, and a higher increase of GST activity with respect to untreated stressed plants (Figure 2A; 24 hours). Higher POX activities in plants grown under drought stress in comparison to optimal conditions were shown in several studies, for example on some tomato varieties (Çelik et al., 2017) and different wheat genotypes (Sairam & Srivastava, 2001). Koleška and colleagues observed a reduction in POX activity of tomato plants, grown in limiting nutrient conditions and treated with a biostimulant, confirming the role of these products in oxidative stress prevention (Koleška et al., 2017).

Moreover, despite GB rising under stress conditions, its significant decrease in content was consistent both under optimal and stress conditions (two‐way ANOVA, no interaction with stress) after ERANTHIS® treatment (Figure 2C; GB). Accordingly, different studies reported a reduction of osmolyte content after biostimulant application under stress conditions (Hasanuzzaman et al., 2022).

In agreement with a number of recent studies related to treated crops grown under abiotic stress, ERANTHIS® decreased the overall plant antioxidant response under drought stress confirming the role of biostimulants in mitigating abiotic stress by oxidative stress prevention (Campobenedetto et al., 2021; Francesca et al., 2022; Hasanuzzaman et al., 2022; Koleška et al., 2017; Macias‐Benitez et al., 2021; Ozfidan‐Konakci et al., 2018; Rasul et al., 2021). Moreover, in a previous published work, ERANTHIS® treated plants showed lower malondialdehyde (MDA) levels, proline content and SOD activity than untreated plants when grown under drought stress conditions (Campobenedetto et al., 2021). The stress mitigation hypothesis was corroborated also by the RNA‐seq results, as described below.

### 3.2 Transcriptomic sequencing data and differentially expressed genes

The Illumina sequencing experiment resulted in 1.731 M raw pair‐ended reads (260,7 Gb, mean length 150 bp) with an average number of 36,1 M reads (PE) for the library. Filtering and trimming operations reduced the reads to 1.711 M (98,84%, Table S1). The total amount of high‐quality sequences was 245,6 Gb, for an average of 35.6 M paired‐end reads per library. Mapping to the tomato genome yielded a total of 1.567,7 M reads, with an average mapping success of 91,6% across all samples.

**FIGURE 3:**
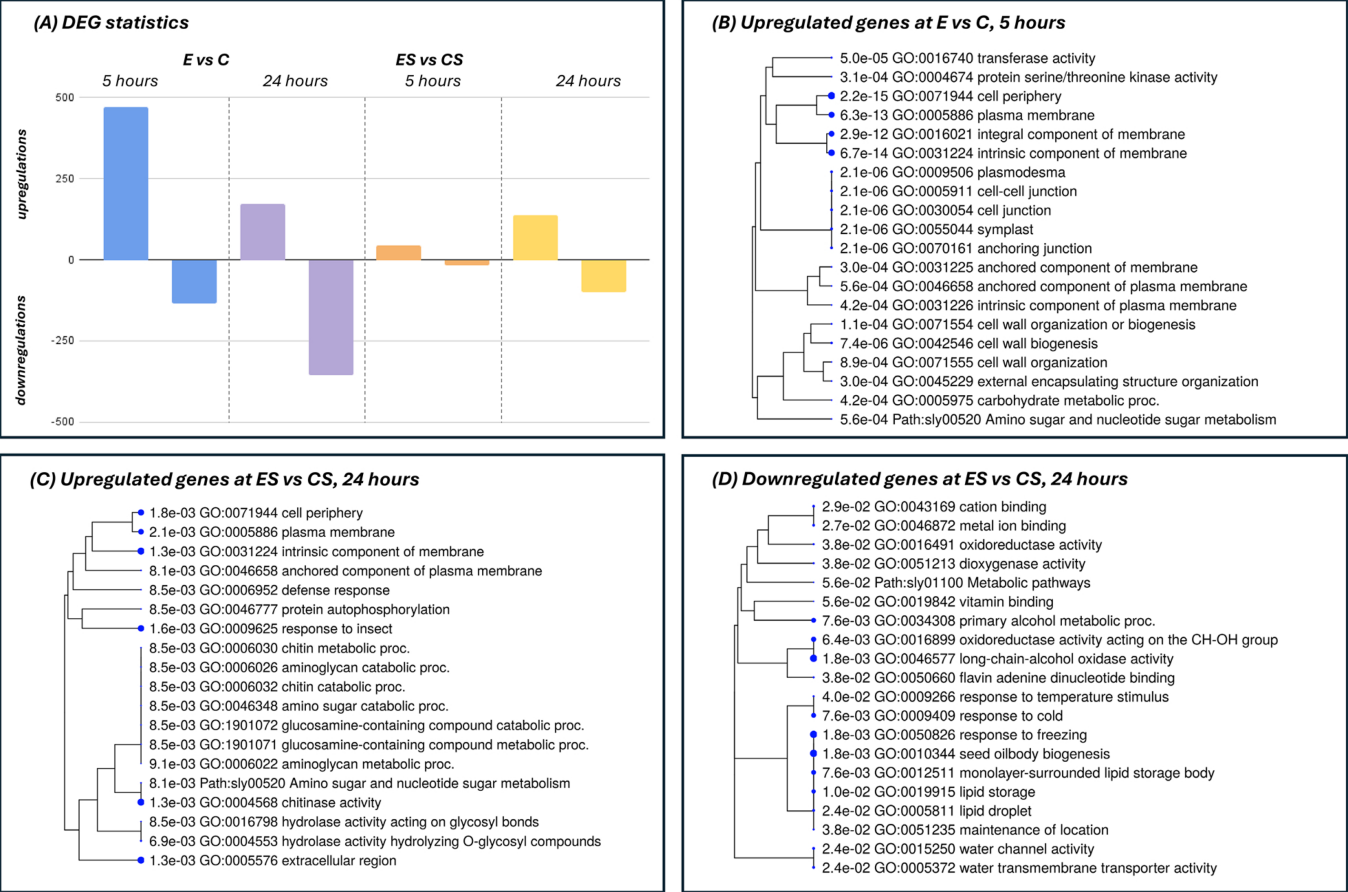
DEG statistics and most representative dendrograms. (A) DEG statistics in terms of number of regulated genes, in the four comparisons (E vs. C at 5 hours in blue and at 24 hours in purple; ES vs. CS at 5 hours in orange and at 24 hours in yellow). In the three other boxes, up and downregulated gene lists enriched with ShinyGO (0.5, FDR = 0.1) in three different conditions are shown: (A) ERANTHIS® unstressed compared with Control unstressed at 5 hours (E vs. C), (B) ERANTHIS® stressed compared with Control stressed at 24 hours (ES vs. CS), (C) ERANTHIS® stressed compared with Control stressed at 24 hours (ES vs. CS). When the branch of the dendrogram ends with a blue dot, it means that the terms associated with that branch are considered biologically relevant and statistically significant.

Transcriptomic analysis showed that ERANTHIS® treatment induces unique gene expression patterns (Figure 3A). Specifically, without water stress, ERANTHIS® unstressed samples (E) exhibited a total of more up‐regulated than down‐regulated genes compared to Control unstressed plants. Specifically, at 5 hours post‐treatment, 469 up‐regulated genes compared to 134 down‐regulated genes were observed, whereas at 24 hours, the number of up‐regulated genes decreased to 170, with 355 down‐regulated, thus showing a shift in gene expression regulation over time. In particular, the greater number of modulated genes at 5 hours after treatment compared to 24 hours indicated that gene regulation processes occur more intensively in the first few hours after treatment. Noteworthy, at 5 hours after treatment, which is an early response after the addition of the biostimulant, under optimal conditions, there was an enrichment in the cell wall organization related GO term (Figure 3; GO:0071555, Fold: 6,15; FDR:0,0009) which was likely influenced by the presence of the biostimulant as shown in Franzoni et al., (2022). Notably, the transcription regulator activity (GO:0140110, (Fold: 2,62; FDR:0,0047)) appeared enriched. A total of 18 up‐regulated transcription factors were observed, among which ERF, WRKY and bHLH were present (Table S2). This indicates an early induction of regulators, likely triggered by the biostimulant treatment (Cocetta et al., 2022), which could drive numerous cellular responses, as reflected in the majority of the observed biochemical changes (Figure 2).

Furthermore, when comparing the ERANTHIS® stressed sample (ES) and the Control unstressed sample (CS), the pattern indicated a higher number of differently regulated genes at 24 hours, compared to 5 hours. Specifically, at 5 hours post‐treatment under stress, we observed 44 up‐regulated genes versus 16 down‐regulated genes. Interestingly, at a later time point of 24 hours, the number of down‐regulated genes increased to 100, with 135 genes being up‐regulated. Therefore, the ratio between up and downregulated genes is higher at 5 hours compared to 24 hours, thus showing a higher downregulation activity as a late response to the biostimulant application under stress conditions. Notably, in the stressed sample after the application of the biostimulant (ES‐24), a gene enrichment related to the oxidoreductase activity (GO:0016491) was present (Table S2). It included two alcohol oxidases as enzymes known to produce hydrogen peroxide (H₂O₂) as a byproduct of their enzymatic activity (Goswami et al., 2013). However, in this experimental condition, these genes are down‐regulated and this is in accordance with the reduced levels of H₂O₂ found compared to the control and likely induced by the biostimulant (Figure 2; Mittler, 2022).

Notably, the water channel activity (GO:0015250) appeared enriched both under optimal and stressed conditions (Figure 3). Under unstressed conditions, a total of five plasma membrane aquaporins (LOC101247747, LOC101248037, LOC101251423, LOC101264605, PIP1‐5) were down‐regulated at 24 hours (Table S2). Two of them (LOC101247747 and LOC101248037), which were also down‐regulated at 5 hours after treatment, belong to the PIP‐2 family, while the others are part of the PIP‐1 subfamily. Under stressed conditions, only two tonoplast aquaporins (LOC101250514, LOC101251154) appeared down‐regulated at 24 hours after treatment. Therefore, the ERANTHIS® treatment induced a specific timing and stress‐dependent regulation of tomato leaf numbers, type, and subfamily of aquaporin transcripts.

Aquaporins are integral membrane proteins that facilitate the transport of water across cell membranes, playing a crucial role in maintaining cellular water homeostasis (Maurel et al., 2008). The observed down‐regulation in response to biostimulants suggests a complex interaction in which these products may enhance water use efficiency or activate alternative water management, thus mitigating the effects of drought (Rouphael & Colla, 2020, Calvo et al., 2014). In accordance with our results, PIP‐1 aquaporin expression was downregulated at 48 hours after a spray treatment based on an *Ascophyllum nodosum* extract in *Solanum lycopersicum* under controlled growth conditions (Baghdadi et al., 2022). Recent studies have highlighted aquaporin's role in modulating leaf water regulation, gas exchanges, redox homeostasis and osmoregulation particularly under stress conditions (Byrt et al., 2023). Under water stress conditions, the presence of ERANTHIS® appears to further modulate aquaporin expression, by downregulating tonoplast aquaporins (TIPs), thus probably acting on leaf water retention, cell turgor pressure and water potential, as previously observed (Campobenedetto et al., 2021).

### 3.3 Multivariate analysis and gene‐enriched sets from clusters identified with Genesis

The k‐means algorithm was applied to the experiment data from the biostimulant treatment under optimal and stress conditions in order to organize the regulated genes into distinct clusters and further support the gene response to the treatment (Figure S1, Figure S2). The two k‐means analyses were separately investigated expressing all the data points in log2FC, specifically normalized. A careful examination of data was done using the Figure of Merit (FOM) algorithm to ascertain the optimal cluster number for division. Specifically, 45 clusters were identified (Figure S1, Figure S2), with a notable variance in gene count. In the experiment without water stress, cluster size ranged from one gene in the smallest cluster (cluster 38) to 1937 genes in the largest one (cluster 7), with an average of approximately 437 genes per cluster. In the experiment with water stress, cluster size ranged from 21 genes in the smallest cluster (cluster 9) to 1963 genes in the largest one (cluster 43), with an average of approximately 437 genes per cluster. Subsequently, a narrowed focus on nine out of the 45 clusters revealed distinct expression patterns in relation to control and ERANTHIS®‐treated samples at different time points.

**FIGURE 4:**
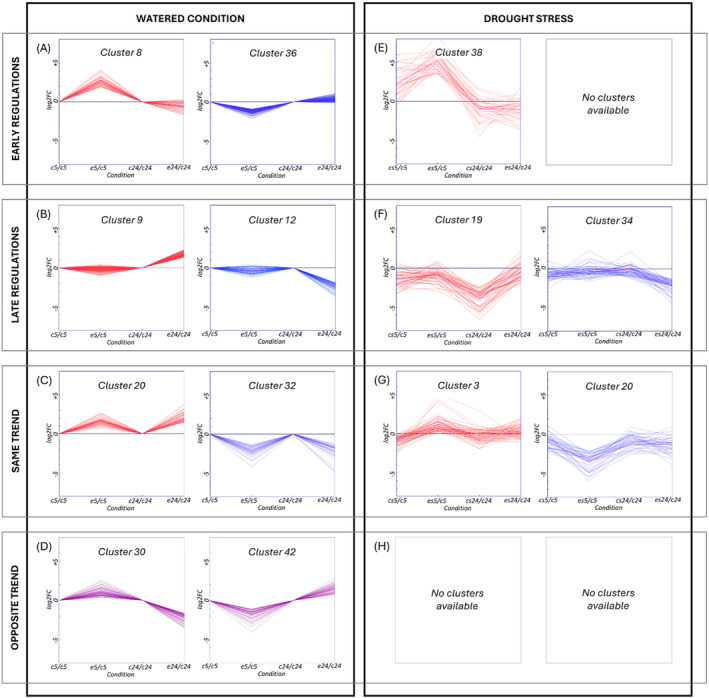
Clusters selected from the k‐means analysis. The diagram shows gene expression trends in specific clusters under different watering conditions. Normal watering conditions: (A) early up‐regulations at 5 hours in Cluster 8 and down‐regulations in Cluster 36; (B) late up‐regulations at 24 hours in Cluster 9 and down‐regulations in Cluster 12; (C) consistent trends at both 5 and 24 hours, with up‐regulations in Cluster 20 and down‐regulations in Cluster 32; (D) opposite trends: Cluster 30 shows up‐regulations at 5 hours and down‐regulations at 24 hours; Cluster 42 exhibits the reverse trend. Drought stress conditions: (E) early up‐regulations at 5 hours in Cluster 38; (F) late up‐regulations at 24 hours in Cluster 19 and down‐regulations in Cluster 34; (G) consistent trends at both 5 and 24 hours: up‐regulation trends in Cluster 3 and down‐regulation trends in Cluster 20; (H) no opposite expressions were observed. For detailed expression data, refer to Figures S1 and S2.

**FIGURE 5:**
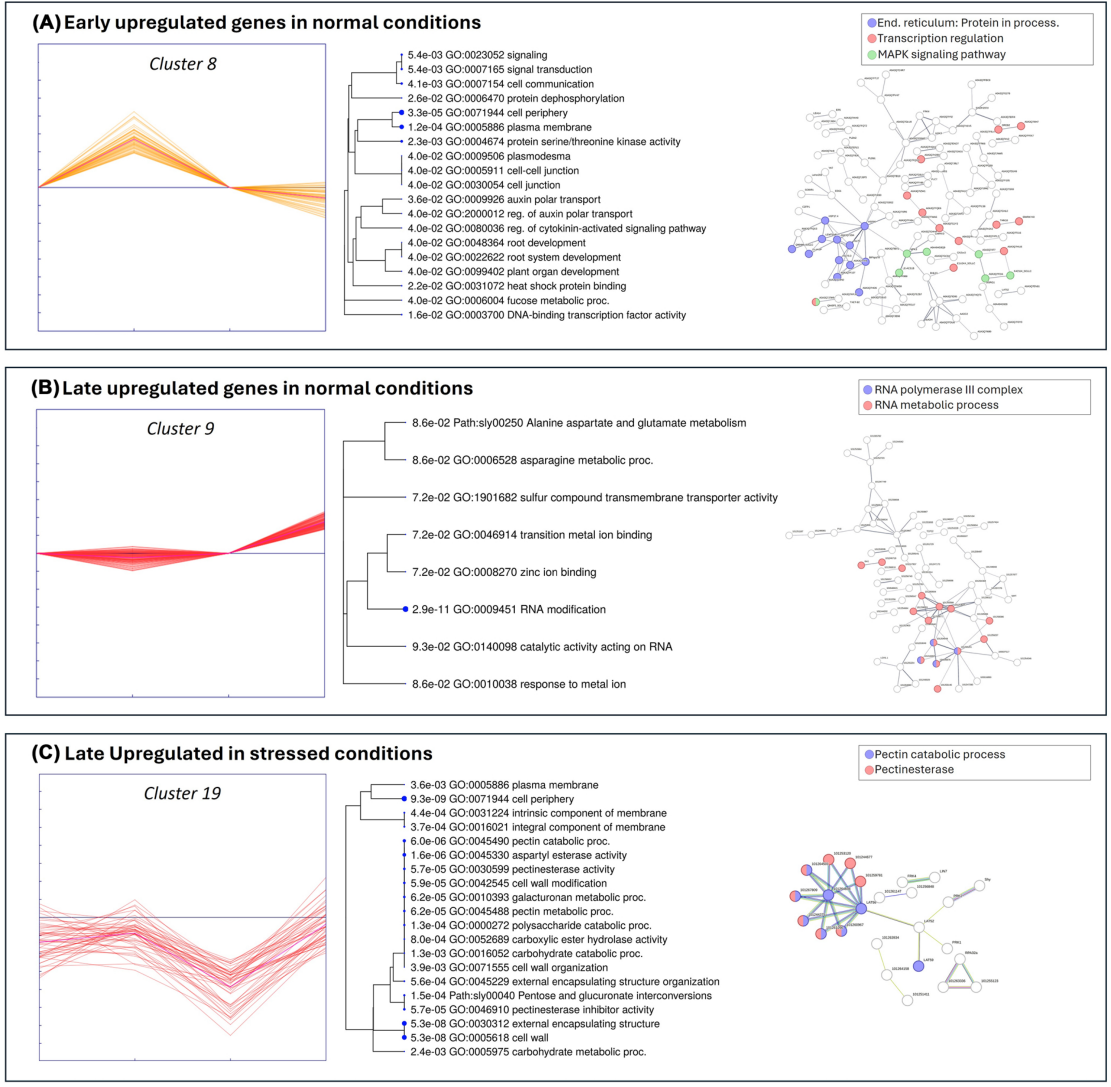
Trends, enrichments and interactions of the most representative clusters. This figure illustrates the trends, enrichments, and interactions for clusters 8, 9, and 19, highlighting their specific behaviors. The enrichment dendrogram (created with ShinyGO 0.8, FDR = 0.1) and the interaction network (generated using STRING with a minimum interaction score of 0.5) combine gene lists from clusters with similar patterns. In panel (A), Cluster 8 shows early up‐regulation in normal conditions. Key pathways include protein processing in the endoplasmic reticulum (blue: sly04141) and transcription regulation (red: KW‐0805). Panel (B) highlights Cluster 9 with late upregulation in normal conditions. Important processes are the RNA metabolic process (red: GO:0016070) and the RNA polymerase III complex (blue: GO:0005666). Panel (C) focuses on Cluster 19, which is late upregulated under stressed conditions. Significant pathways include the pectin catabolic process (blue: GO:0045490) and pectinesterase activity (red: PF01095).

In both experiments (water stress vs. optimal conditions), numerous clusters exhibited gene expression behaviors that remained unchanged at the two time points (5 and 24 hours), with respect to the presence/absence of the water stress and/or the biostimulant treatment. Only the clusters that demonstrated major deviations were emphasized (Figures 4, 5). The cluster analysis highlighted a complex network of genes influenced by the biostimulant treatment. The analysis suggests that the biostimulant application could modulate gene expression through a series of regulatory networks coordinating gene expression in presence/absence of water stress. These findings, along with the functional annotations, are extensively documented in Table S3 and S4.

#### 3.3.1 ERANTHIS® modulates metabolic and signaling gene pathways in optimal conditions

The clustering analysis aimed to examine the response of tomato plants at the molecular level, in terms of changes in gene expression, after the application of ERANTHIS®. Following the treatment, several clusters displayed a variation in gene expression (Figure 3). Some of them showed significant positive changes only at 5 hours after treatment (clusters 8, 18, 26, 28, 37), or negative changes (clusters 21, 36), while others responded later to ERANTHIS® treatment after 24 hours with an increase in gene expression (clusters 9, 25, 29, 43) or with a decrease in gene expression (clusters 5, 12, 41). Clusters 3, 4, 11, 20 and 22 were augmented at both experimental times, indicating a sustained response to the biostimulant, while genes in Clusters 2, 32, 38 and 39 were mitigated at both experimental times. Clusters 30, 35, 44 showed contrasting behaviors (increased at 5 hours and decremented at 24 hours) and genes in Clusters 19, 42 had an opposite behavior (decreased at 5 hours and increased at 24 hours).

In examining these enhanced responses of tomato plants to the application of the biostimulant, significant enrichments were observed in hormone regulation pathways such as auxin and cytokinin signaling, which play pivotal roles for plant growth (GO:2000012, GO:0080036, GO:0080037). This was complemented by enhancements in membrane‐associated signaling and metabolic adaptations (GO:0031234, GO:0019897), aligning with the biostimulant role in boosting plant growth and metabolic capacity (Calvo et al., 2014; Lucini et al., 2015; Povero et al., 2016). Notably, transcription factors like WRKY40 (LOC100301944) and NAC domain‐containing proteins (LOC101253916, LOC101264084, LOC101264451, 101263872) were early up‐regulated, indicating their roles in stress responses and hormonal signaling (Chen et al., 2010; Puranik et al., 2012). Also in the late responses, significant enrichments were observed in amino acid metabolism and nutrient uptake processes (GO:0006528, GO:0062014, GO:1901682, GO:0008514), suggesting prolonged effects of the biostimulant on plant nutrition and health (Bulgari et al., 2019; Calvo et al., 2014). Additionally, the constant modulation of genes related to the plasma membrane and microtubule structures (GO:0046658, GO:0031225, GO:0005874) across different time points highlights the biostimulant influence on cytoskeletal architecture and membrane stability, essential for cell division and signaling (Baltazar et al., 2021).

##### Early Genes‐ Biostimulant effects in optimal conditions

At 5 hours post‐ treatment, there was a significant up‐ regulation of genes involved in hormone regulation and metabolic processes (Table S3). Key enriched pathways include auxin and cytokinin signaling (GO:2000012, GO:0080036, GO:0080037), crucial for plant growth and enhancements in plasma membrane and secondary metabolic pathways (GO:0031234, GO:0019897, GO:0043455), suggesting modifications in membrane‐associated signaling and metabolic acclimatization (Calvo et al., 2014; Povero et al., 2016). Among the auxin‐response‐related genes, the up‐regulation of *SAUR* family genes (LOC101252288, LOC104645438, LOC101268293, LOC101258256, LOC101255313, LOC101266965), which belong to GO:0042221 (response to chemicals), may be linked not only to plant growth regulation but also to improved tolerance to salt and drought stress (Stortenbeker & Bemer, 2019). Moreover, the up‐regulation of the MAPK signaling pathway (Path:sly04016, Figure 5A) and the biosynthesis of secondary metabolites (Path:sly01110) align with the known benefits of biostimulants in boosting plant metabolic functions (Lucini et al., 2015). Noteworthy, transcription factors such as WRKY40 (LOC100301944), NAC proteins (LOC101253916, LOC101264084, LOC101264451, LOC101263872), MYB44 (LOC101249225), ERF027 (LOC101260455), and bHLH106 (LOC101266278) were also up‐regulated, indicating their roles in stress responses, hormone signaling, and developmental processes (Figure 5A; Chen et al., 2010; Jung et al., 2008; Müller & Munné‐Bosch, 2015).

Conversely, certain genes exhibited notable down‐regulation at 5 hours post‐treatment within Clusters 21 and 36, associated with the xyloglucan biosynthetic process (GO:0009969) and dolichol metabolic process (GO:0019348), which may influence cell wall composition and lipid metabolism (Table S3). Additional pathways such as lipid storage (GO:0019915) and nucleosomal DNA binding (GO:0031492) were also under‐expressed, reflecting the biostimulant impact on energy storage and gene expression regulation (Campobenedetto et al., 2020). Down‐regulated transcription factors included ASR3 (LOC101267286), GATA16 (LOC101266764), and MYB41‐like (LOC101252700), which are implicated in nutrient and lipid metabolism pathways, potentially optimizing energy storage and utilization (Kosma et al., 2014; B. Li et al., 2015; Ran et al., 2022).

##### Late Genes‐ Biostimulant effects in optimal conditions

At 24 hours after treatment, the late response to ERANTHIS® in clusters 9, 25, 29, and 43 revealed significant enrichment in the asparagine metabolic process (GO:0006528) and the negative regulation of small molecule metabolic processes (GO:0062014), suggesting an influence on amino acid metabolism that could enhance plant growth and stress resilience (Bulgari et al., 2019). The identification of sulfur compounds and organic anion transmembrane transporter activities (GO:1901682, GO:0008514) may implicate a role in nutrient uptake and transport, essential for plant nutrition (Calvo et al., 2014). In particular, two up‐regulated ω‐amidase loci (101257877, 101257275) were observed (Table S3). They are known to be key enzymes in the generation of 2‐hydroxy‐5‐oxoproline (2HOP), acting as a signal of ammonium assimilation, promoting enhanced growth, yields and stress tolerance (Unkefer et al., 2023). Additionally, an enrichment in the alanine, aspartate, and glutamate metabolism pathways (Path:sly00250) was observed (Figure 5), thus suggesting a possible link to the biostimulant high content in peptides and to the regulation in proline level (Figure 2). The up‐regulation of genes involved in RNA modification (GO:0009451) and catalytic activity acting on RNA (GO:0140098) suggests impacts on RNA metabolism (Figure 5B), as observed in other biostimulant studies (Campobenedetto et al., 2020). The response to metal ions (GO:0010038) and specific ion binding activities (GO:0008270, GO:0046914) also suggests modulation of ion homeostasis, vital for plant adaptation to environmental stresses (Povero et al., 2016). Notable transcription factors such as ERF4 and TCP22 were up‐regulated, potentially enhancing plant stress resilience and regulating growth and development (Danisman, 2016; Viola et al., 2023; G. Zhang et al., 2009).

Concurrently, a general down‐regulation was observed in clusters 5, 12, and 41, particularly affecting genes associated with transmembrane receptor protein serine/threonine kinase activity (GO:0004675) and kinase activity (GO:0019199), suggesting a decrease in specific growth and stress response signaling pathways. This modulation suggests plants fine‐tuning in their cascade signaling in response to the biostimulant as observed in Della Lucia et al., (2022), which mainly occurred at 5 hours after treatment under optimal conditions. The decrease in transmembrane signaling receptor activity (GO:0004888) and signaling receptor activity (GO:0038023) points towards a broad impact of the biostimulant on the plant ability to perceive and respond to external signals. The enrichment in pathways related to the cell periphery and plasma membrane (GO:0071944, GO:0005886) further underscores the biostimulant impact on membrane‐associated processes, potentially affecting nutrient uptake, signal transduction, and environmental interactions (Della Lucia et al. (2022). Notably, the down‐regulation of ERF‐H2 and ERF‐H14, components of the ethylene signaling pathway, suggests a nuanced role of the biostimulant in plant stress responses and development (Müller & Munné‐Bosch, 2015).

##### Constant Genes‐ Biostimulant effects in optimal conditions

The constant response to ERANTHIS® treatment, observed in clusters 3, 4, 11, 20, and 22, involves the enrichment of GO terms related to the anchored component of the plasma membrane (GO:0046658, GO:0031225) and microtubule structures (GO:0005874). This suggests an influence on cytoskeletal architecture and membrane stability, crucial for cell shape, division, and signaling (Baltazar et al., 2021). Interestingly, microtubule dynamics can be directly related to plant adaptations to osmotic stressors (Chun et al., 2021). Additionally, pathways related to plant‐pathogen interactions (Path:sly04626) and phenylpropanoid biosynthesis (Path:sly00940) were enriched, indicating a preparative role of ERANTHIS® in enhancing plant defense mechanisms and secondary metabolite production (Baltazar et al., 2021). Similarly, seaweed extract from the brown algae *Ascophyllum nodosum* up‐regulated priming genes in tomato associated with plant‐pathogen interactions. In addition, it temporarily induced ROS production, as seen with H₂O₂ levels in our experiments (Figure 2; Islam et al., 2021). Moreover, the modulation of signaling pathways through protein serine/threonine kinase activity (GO:0004674) and protein phosphorylation (GO:0006468) highlights the pivotal role of the biostimulant in orchestrating the plant signaling responses (Nephali et al., 2020). Transcription factors such as WRKY22 (LOC101261749) and MYB62 (LOC101244584), involved in regulating phenylpropanoid biosynthesis, contribute to secondary metabolism and stress responses, influencing biostimulant effects on metabolite production (Dubos et al., 2010; Rushton et al., 2010). Ethylene‐responsive transcription factor ERF‐A2 (LOC101267589) modulates ethylene signaling, potentially boosting plant resilience (Müller & Munné‐Bosch, 2015).

Concurrently, the constant negative response in clusters 2, 32, 38, and 39 relates to the down‐regulation of genes associated with peptide:proton symporter activity (GO:0015333), suggesting a modulation in active transport mechanisms for peptides across the plasma membrane, which could influence nutrient uptake and nitrogen assimilation. This specific modulation might reflect a strategic adjustment to optimize energy expenditure in response to the biostimulant presence (Michalak et al., 2022). Additionally, the reduction in acyltransferase activity (GO:0016747) points towards alterations in lipid metabolism, potentially affecting membrane lipid composition and signaling lipid molecule synthesis. The observed decrease in genes related to the plasma membrane (GO:0005886) and cell periphery (GO:0071944) further underscores the impact of ERANTHIS® on membrane‐associated processes, possibly influencing cell signaling, transport, and interaction with the external environment. The down‐regulation of specific transcription factors such as WRKY26 (LOC101260537), MADS‐box protein SOC1 (LOC544075), and Jasmonate‐responsive ERF 4 (JRE4) indicates a modulation of plant physiological processes, particularly affecting/reducing peptide transport, growth regulation, and lipid metabolism (Müller & Munné‐Bosch, 2015; Rushton et al., 2010; Smaczniak et al., 2012), to maintain cellular integrity under the biostimulant influence (Rushton et al., 2010).

##### Contrasting Genes‐ Biostimulant effects in optimal conditions

The observed up‐regulation of genes involved in specific pathways at 5 hours, followed by their subsequent mitigation at 24 hours, suggests a temporal regulation of these responses, providing insights into the timing of ERANTHIS®‐mediated effects on plant physiology. The contrasting response to the biostimulant treatment in clusters 30, 35, and 44 shows an enrichment of GO/KEGG terms related to strigolactone metabolic and biosynthetic processes (GO:1901600, GO:1901601), thiol oxidase activity (GO:0016972), pectinesterase activity (GO:0030599) and various galactosyltransferase activities (e.g., GO:0048531 beta‐1,3‐galactosyltransferase activity). This suggests a significant role of the biostimulant in influencing hormone signaling pathways, redox homeostasis, and cell wall modification (Tripathi et al., 2024). Interestingly, what is observed for the thiol synthase activity (GO:0016972) could be also related to biochemical data (Figure 2C). Indeed, the modulation of the ERV1 (LOC 101263280) gene was observed. This gene is involved in thiol oxidation, so its up‐regulation leads to the decrease of free thiols, because of their conversion in disulfide bonds. The upregulation at 5 hours and mitigation at 24 hours could explain the decrease of thiols at both time points, compared to the control (Figure 2C). From a biochemical point of view, the use of the biostimulant might induce a stress response or a signaling pathway that up‐regulates ERV1 expression initially (at 5 hours), leading to increased oxidation of thiols and thus a decrease in their concentration. By 24 hours, the system might have adapted or compensated, leading to a mitigation of ERV1 expression, but the thiol levels could still be lower than the control due to the initial oxidative activity (Tu & Weissman, 2004; Sevier & Kaiser, 2008). Additionally, the enrichment in processes such as proton export across the plasma membrane (GO:0120029), lactone biosynthetic process (GO:1901336), and pectin catabolic process (GO:0045490) underscores the biostimulant potential in modulating nutrient uptake, secondary metabolite production, and cell wall restructuring. These findings align with previous studies highlighting the role of biostimulants in modulating similar pathways for improved plant growth and stress resilience (Calvo et al., 2014; du Jardin, 2015). Regarding cell wall modification, the regulation of genes involved in pectin metabolism, like *pectin methylesterase* (LOC101261266) and *pectinesterase 1‐like* (LOC101244375) could be useful for acclimatization in case of environmental stress occurence (Du et al., 2020).

The analysis of genes mitigated at 5 hours and incremented at 24 hours by ERANTHIS® in clusters 19 and 42 under optimal watering conditions reveals significant enrichment in GO/KEGG terms associated with DNA replication and repair, cell cycle regulation, and signal transduction pathways (Ertani et al., 2017). Notably, the enrichment of GO terms such as MCM complex (GO:0042555), THO complex (GO:0000347), and plus‐end‐directed microtubule motor activity (GO:0008574) underscores the impact of the biostimulant on the fundamental processes of cell division and genome stability. The observed enrichment in pathways related to pyrimidine metabolism (e.g., dUMP metabolic process GO:0046078, dUTP metabolic process GO:0046080) and lipid glycosylation (GO:0030259) suggests a likely influence of the biostimulant on nucleotide synthesis and membrane dynamics, essential for cell growth and response to environmental stimuli (Rathore et al., 2022).

#### 3.3.2 ERANTHIS® modulates stress‐related pathways in stressed conditions

This experimental step aimed to examine the response of tomato plants subjected to water stress after applying the biostimulant. In this condition, specific genes displaying an increase or mitigation behavior and involved in specific pathways, were identified (Figure 4). These pathways are essential for plant acclimation to environmental stresses, and were in early activated clusters (9, 30, and 38), associated with MAPK signaling pathway and plant and cell wall strengthening (sly04016, GO:0071555). They included genes like MAPKKK18‐like (LOC101258387) and transcription factors such as NAC32‐like (LOC101244243) and MYB62‐like (LOC101266622), which are crucial for orchestrating stress responses and enhancing drought resilience (Butt et al., 2017; Jonak et al., 1996; Majeed et al., 2023; Moustafa et al., 2014). Late responses under stress conditions (clusters 6, 19, and 32) further emphasized the role of the biostimulant in reinforcing cell wall architecture (GO:0005618) and modulating ethylene‐mediated signaling pathways through transcription factors like EREB, enhancing the plant ability to cope with prolonged water stress (Le Gall et al., 2015; Müller & Munné‐Bosch, 2015). The constant modulation of genes (clusters 3) related to cell wall biogenesis and transcriptional regulation across both early and constant responses in stressed conditions (GO:0009832, GO:0071554, GO:0055029, GO:0000428) underscores the biostimulant impact on plant structural integrity and acclimation to environmental stresses (Bulgari et al., 2015; Calvo et al., 2014). These findings collectively showed the role of the biostimulant in enhancing plant growth, resilience, and acclimation through a complex modulation of transcriptional and hormonal pathways.

##### Early Genes‐ Biostimulant effects in water stress conditions

At 5 hours post‐treatment, significant changes were observed in clusters 9, 30, and 38. In cluster 9, genes associated with actin filament organization, assembly, and formation (GO:0051017), and MAPK signaling pathway‐plant (sly04016) were notably up‐regulated. This included the MAPKKK18‐like gene (LOC101258387), which plays a role in the mitogen‐activated protein kinase pathway, critical for mediating cold and drought stress signaling in plants (Jonak et al., 1996; Majeed et al., 2023; Moustafa et al., 2014). Additionally, transcription factors such as NAC transcription factor 32‐like (LOC101244243) and MYB62‐like (LOC101266622) were up‐regulated, suggesting their roles in orchestrating the plant response to water stress, potentially enhancing osmotic stress tolerance (Butt et al., 2017). The Homeobox‐leucine zipper protein HDG1 (LOC101266128) also indicates the involvement of HD‐Zip transcription factors in improving water use efficiency and stress tolerance (Y. Li et al., 2022). This gene, indeed, promotes the production of cuticles on leaves, potentially limiting, together with other genes involved in cell wall organization, water loss, thus reducing the negative effects of drought stress in plants treated with the biostimulant (Wu et al., 2011).

Cluster 30 showed up‐regulation of genes related to nucleosome organization and assembly, water transmembrane transporter activity (GO:0006833), biogenesis (GO:0071840), and cell wall organization (GO:0071555), including genes like *pectinesterase‐like* (LOC101260787), *cellulose synthase‐like protein D5* (LOC101262856), and x*yloglucan endotransglucosylase‐hydrolase* (*XTH3*) which are crucial for modifying cell wall architecture to enhance water retention and structural integrity under stress (Cosgrove, 2005). Interestingly, changes in cell wall organization and water transmembrane activity could be linked to a previous experiment in which ERANTHIS® treatment reduced stem water potential loss under mild water stress conditions (Campobenedetto et al., 2021). In the end, cluster 38 also included up‐regulated genes involved in the formation of plant structural polymers, like the F‐box/kelch‐repeat protein (LOC101267757). This gene interacts with PAL (Phenylalanine ammonia lyase) genes, altering the content of phenylpropanoids, important molecules involved in plant polymer formation (X. Zhang et al., 2013). In the same cluster, the up‐regulation of thaumatin‐like protein (LOC101248168) could be related to the enhancement of stress tolerance in treated plants (He et al., 2021).

Conversely, cluster 20 exhibited a down‐regulation in gene expression, particularly in genes associated with oxidoreductase activity (GO:0016722). This includes genes like *laccase* (LOC101262074), *geraniol 8‐hydroxylase‐like* (LOC101262074), and *inositol 2‐dehydrogenase* (LOC101260461), which are involved in managing oxidative stress, modifying plant structural components, and osmoprotectant accumulation. The down‐regulation of laccase might indicate a modulation of lignin biosynthesis, affecting plant rigidity and water transport efficiency, potentially optimizing water use and retention under drought conditions (Ranocha et al., 2002). The observed decrease in expression of these genes might indicate that the biostimulant preconditions plants to tolerate oxidative stress by altering their baseline oxidative state, thus reducing the need for active response during stress, leading to a more efficient physiological acclimatization to drought stress (Calvo et al., 2014). At the same time, it is interesting to note the downregulation of the probable galacturonosyltransferase (GAUT)‐like gene (LOC101257137). As reported by Godoy and colleagues, mutant tomato plants with silenced GAUT show a higher content of water in leaves (De Godoy et al., 2013), supporting the idea expressed above, whereby the leaves of the treated plants would be able to retain water more than the control ones.

##### Late Genes‐ Biostimulant effects in water stress conditions

At 24 hours post‐treatment, significant changes were observed in clusters 6, 19, and 32. Cluster 6 showed an increase in genes related to the integral component of the plasma membrane (GO:0005887). In this cluster, there are also different genes strictly related to stress tolerance, like *probable polyol transporter 6* (LOC101247786) whose overexpression increases the entry of polyols into the cell, increasing drought stress tolerance (Petrozza et al., 2014) and *putative glycine‐rich cell wall structural protein 1* (LOC101265562) that increases stress tolerance when up‐regulated. Moreover, the up‐regulation of *beta‐glucosidase BoGH3B‐like* (LOC101250040) is related to proline content (Gutiérrez et al., 2023). The increase of this osmolyte was also observed from a biochemical point of view at 24 hours after the biostimulant treatment (Figure 2B). Cluster 19 exhibited enrichments in cellular component biogenesis (GO:0071840), while cluster 32 highlighted several genes associated with the cell wall (GO:0005618), involved in catabolism of pectins (Figure 5C). This suggests a continued focus on changing cell wall architecture in response to prolonged water stress, potentially enhancing the plant resilience to drought (Le Gall et al., 2015). Indeed, as for early genes, we found some genes involved in enhancing the leaf structure, helping plants to cope with drought stress. In particular, the up regulation of *probable glycosyltransferase* (LOC101255224) in cluster 6 is related to pectin biosynthesis, while in cluster 32 the up‐regulation of *fatty acyl‐CoA reductase 1‐like* (LOC101255165), *COBRA‐like protein 10* (LOC101256898) and *fructokinase* (FRK4) could be related to cuticle waxy layer formation and cellulose deposition (De Godoy et al., 2013) thus affecting leaf permeability.

Moreover, the ethylene‐responsive element‐binding protein (EREB) from the ERF family in cluster 32 was up‐regulated, indicating the activation of ethylene‐mediated signaling pathways which likely enhance the plant ability to cope with water stress through growth modulation and stress defense mechanisms (Müller & Munné‐Bosch, 2015). Additionally, the presence of a gene similar to MAPKKK18 (LOC101249831) in cluster 6 suggests a sustained role of SAPK signaling in mediating the plant response to prolonged water stress (Wasternack & Hause, 2013). The up‐regulation of genes such as *mitogen‐activated protein kinase kinase kinase 18‐like* (LOC101258387) and cell wall modification enzymes (e.g., *pectinesterase‐like* LOC101260787, *cellulose synthase‐like protein D5* LOC101262856) further supports the activation of critical adaptive responses (Figure 5C). Other transcription factors, including GATA *transcription factor 16* (LOC101266764), EREB, and *Homeobox‐leucine zipper protein* HDG1 (LOC101266128), appeared later in the analysis. In rice, GATA16 acts as a transcriptional suppressor and has been observed to be induced by cold and ABA treatments, but repressed by drought. However, its expression was promoted in the presence of the biostimulant (H. Zhang et al., 2021).

Few enrichments were observed in down‐regulated late genes (cluster 34). This minimal down‐regulation at 24 hours post‐treatment underlines the sustained up‐regulatory response facilitated by the biostimulant in adapting to water stress conditions. The focus remains predominantly on stress response mechanisms through the modulation of multiple genes involved in actin filament network formation (GO:0051639) and the regulation of auxin polar transport (GO:2000012), facilitating an adaptive response to environmental stresses (Roomi et al., 2018).

##### Constant Genes‐ Biostimulant effects in water stress conditions

The presence of genes constantly up‐regulated in water stress conditions following biostimulant treatment (clusters 3) showed an enrichment in genes related to cell wall biogenesis and organization (e.g., GO:0009832 plant‐type cell wall biogenesis, GO:0071554 cell wall organization or biogenesis), indicating and influence on plant structural integrity and growth. For example, the up regulation of *chitinase 2‐like* (LOC101265800) and *serine carboxypeptidase‐like 42* (LOC101262410) is related to the increase of drought stress tolerance. Indeed, they are more expressed in tolerant species than in sensitive ones (Xu et al., 2021; Yu et al., 1998). Moreover, the overexpression of *protein ECERIFERUM 1* (LOC101268663) is again related to cuticle permeability, making treated plants more capable of retaining water at leaf level than control (Bourdenx et al., 2011). Additionally, enrichments in terms associated with RNA polymerase complexes (e.g., GO:0055029 nuclear DNA‐directed RNA polymerase complex, GO:0000428 DNA‐directed RNA polymerase complex) suggest an impact on transcriptional regulation, potentially enhancing the plant responsiveness to environmental stimuli. The modulation of hormone‐mediated signaling pathways (e.g., GO:0009755 hormone‐mediated signaling pathway) and cellular responses to hormones (GO:0032870 cellular response to hormone stimulus) further highlight the biostimulant role in fine‐tuning hormonal responses, crucial for plant development and stress resilience. These findings align with previous research demonstrating the capacity of yeast‐ and algae‐based biostimulants to enhance plant growth and stress tolerance by altering transcriptional and hormonal pathways (Bulgari et al., 2015; Calvo et al., 2014), offering valuable insights for leveraging biostimulants in agriculture to improve crop performance under varied water conditions.

## 4 CONCLUSIONS

In conclusion, our study presents novel insights on the mode of action of ERANTHIS® at its third application which is known to be the most effective on tomato plant performance.

**FIGURE 6:**
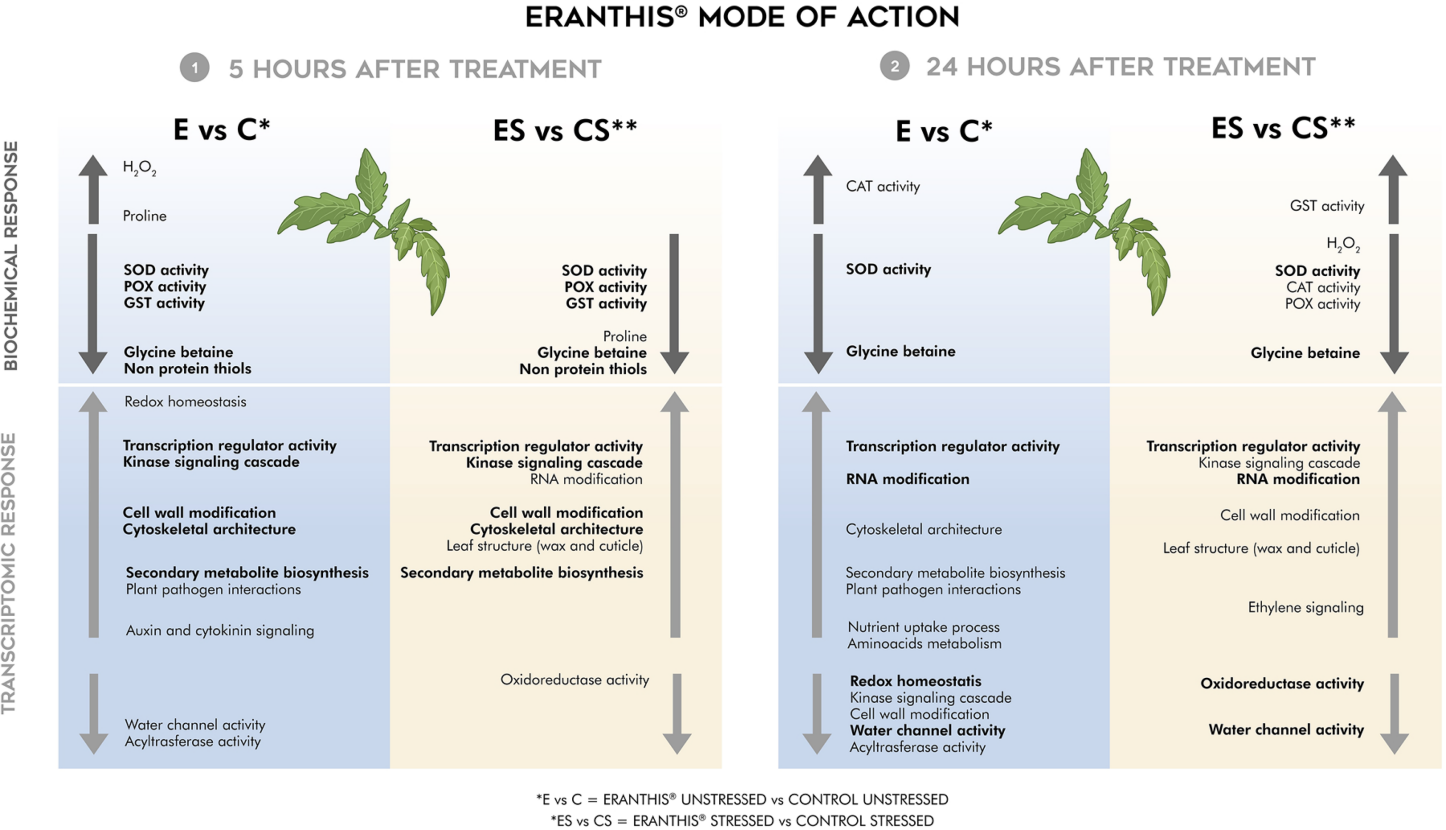
ERANTHIS® mode of action. ERANTHIS® regulation of biochemical responses in the upper rectangles and transcriptomic responses in the lower rectangles at 5 hours (early) and 24 hours (late) after treatment under well‐watered (light blue) and stress (light yellow) conditions. The upwards arrows refer to upregulated responses, the downwards arrows refer to downregulated responses, related to “E vs C" and “ES vs CS” conditions. The same line corresponds to the same type of regulation, while the bolded responses are those common between well‐watered and stress conditions at the same sampling time. CAT = Catalase; GST = Glutathione S‐Transferase; POX = Peroxidase; SOD = Superoxide Dismutase.

The monitoring of its influence on leaf oxidative status and transcriptomic regulation of tomato plants grown under both unstressed and mild water stress conditions at early (5 hours after treatment) and late times (24 hours after treatment) shed a light on the consistency or differences in tomato responses (Figure 6).

Under optimal conditions, the application of ERANTHIS® on tomato plants led to a strategic modulation of the analyzed responses, suggesting that it acts as a priming agent that enhances the plants' stress resilience (i.e.: eu‐stressor). As for the oxidative response, ERANTHIS® treatment induced a H_2_O_2_ rise signaling after 5 hours and modulated ROS non enzymatic and enzymatic scavengers in a timing specific way. Indeed, proline level and CAT activity increased at 5 and 24 hours after treatment, respectively, whereas a reduction in POX and GST activity together with NPSH level occurred only at 5 hours after treatment. ERANTHIS® application also reduced SOD activity and glycine betaine content at both sampling times independently of water stress occurrence, thus suggesting a different regulation of the antioxidant and osmotic responses induced by the product.

As for transcriptomics under unstressed conditions, ERANTHIS® treatment increased the expression of genes related to transcription regulatory activity at both time points and genes related to RNA modification at 24 hours post treatment, thus changing the overall gene expression and possibly acting on post‐transcriptional modulation. As a relevance of post‐treatment signaling occurrence, an early increase and a later decrease in the expression of genes related to kinase signaling cascade, redox homeostasis and cell wall modification is observed. The treatment constantly modulated the expression of specific metabolic pathways related to plant defense mechanisms like plant pathogen interaction and secondary metabolite biosynthesis, and stress resilience pathways around cytoskeletal architecture and water channel activity. Moreover, it increased the expression of genes involved in growth regulation, by early acting on hormone signaling via auxin and cytokinin, and later acting on nutrient uptake processes and amino acid metabolism. This multifaceted approach can prepare the plant for potential environmental challenges but can also promote overall growth.

Under water stress conditions, tomato oxidative status and transcriptomic responses after ERANTHIS® application confirmed the biostimulant role in mitigating drought stress. As for oxidative status, H_2_O_2_ rising induced by stress is decreased by the treatment after 24 hours and ROS enzymatic and non‐enzymatic scavenging is specifically modulated by the treatment. At 5 hours after treatment, antioxidant enzyme activity regulation is similar to that observed in treated unstressed plants. Moreover, ROS scavenging enzyme activities and non‐enzymatic antioxidant levels showed a dominant decreasing trend with respect to untreated stressed plants at 24 hours and 5 hours after treatment, respectively.

As for transcriptomics, ERANTHIS® showed to modulate the expression of many of the metabolic pathways regulated under optimal conditions, together with the upregulation of genes involved in ethylene signaling at 24 hours after treatment, and leaf structure impermeability at both time points. These gene expression patterns together with the less altered oxidative status with respect to untreated stressed plants can explain how ERANTHIS® mitigates water stress.

These overall results suggest that the selected timings and the adopted RNA‐seq data analysis techniques, by either monitoring differentially expressed genes (DEGs) or groups of co‐regulated genes (k‐means clustering), can be a valid future approach to provide further information about biostimulant modes of action. Future works will be aimed to discriminate the role of ERANTHIS® formulation components aka. brown algae extracts, yeast extracts and selected peptidic‐sources, in modulating its mode of action. Moreover, further research is needed, particularly functional studies of the genes selected through transcriptomic analysis. This will be essential to fully elucidate the mechanisms by which biostimulants confer stress tolerance. Additionally, optimizing the application of biostimulants in agriculture will help to improve plant resilience and crop productivity, especially in an increasingly variable climate and water‐limited environments.

## AUTHOR CONTRIBUTIONS

V.C., C.C., C.M.B. conceived and designed the experiments. C.C. and E.M. grew plants and collected plant material. E.M. performed biochemical experiments. P.C. and A.A. analyzed RNA‐seq data. P.C., A.A., C.C., C.A. prepared figures and the manuscript draft. All authors revised and approved the final version of this manuscript.

## CONFLICT OF INTEREST STATEMENT

V.C., C.C. and C.A are employed by Green Has Italia S.p.A. The remaining authors declare that the research was conducted in the absence of any commercial or financial relationships that could be considered as a potential conflict of interest.

## Supporting information


**Figure S1.** Clusters in optimal conditions ‐ Clustering analysis conducted with Genesis, further normalization of log2FC. A partitional clustering analysis algorithm (k‐means) was used, which allows a set of objects to be divided into 45 groups on the basis of their trends on four comparison points: c5/c5, e5/c5, c24/c24 and e24/c24. The analyzed clusters in which the biostimulant induces a reduction of the expression at 5 or 24 hours are shown in blue, the clusters showing increases in the expression in red, the clusters containing genes with opposite trends between the 5 and the 24 hours sampling in purple. Lastly, in gray all clusters that were not selected for enrichment analysis.

**Figure S2.** Clusters in stressed conditions ‐ Clustering analysis conducted with Genesis, further normalization of log2FC. A partitional clustering analysis algorithm (k‐means) was used, which allows a set of objects to be divided into 45 groups on the basis of their trends on four comparison points: cs5/c5, es5/c5, cs24/c24 and es24/c24. The analyzed clusters in which the biostimulant induces a reduction of the expression at 5 or 24 hours are shown in blue, the clusters showing increases in the expression in red. Lastly, in gray all clusters that were not selected for enrichment analysis.

**Table S1.** RNAseq reads statistics.

**Table S2.** DEGs in optimal water‐stress conditions and related GO enrichments.

**Table S3.** GO enrichments in optimal conditions.

**Table S4.** GO enrichments in water stress conditions.

## Data Availability

Our BioProject and associated RNA‐SEQ metadata are available at https://www.ncbi.nlm.nih.gov/bioproject/PRJNA1137122.
