# Supplementary figures and images for: Antioxidant activity and comparative RNA‐seq analysis support mitigating effects of an algae‐based biostimulant on drought stress in tomato plants
Source: Physiol Plant. 2024 Dec 20;176(6):e70007. doi: 10.1111/ppl.70007 (PMC11659800; doi:10.1111/ppl.70007)

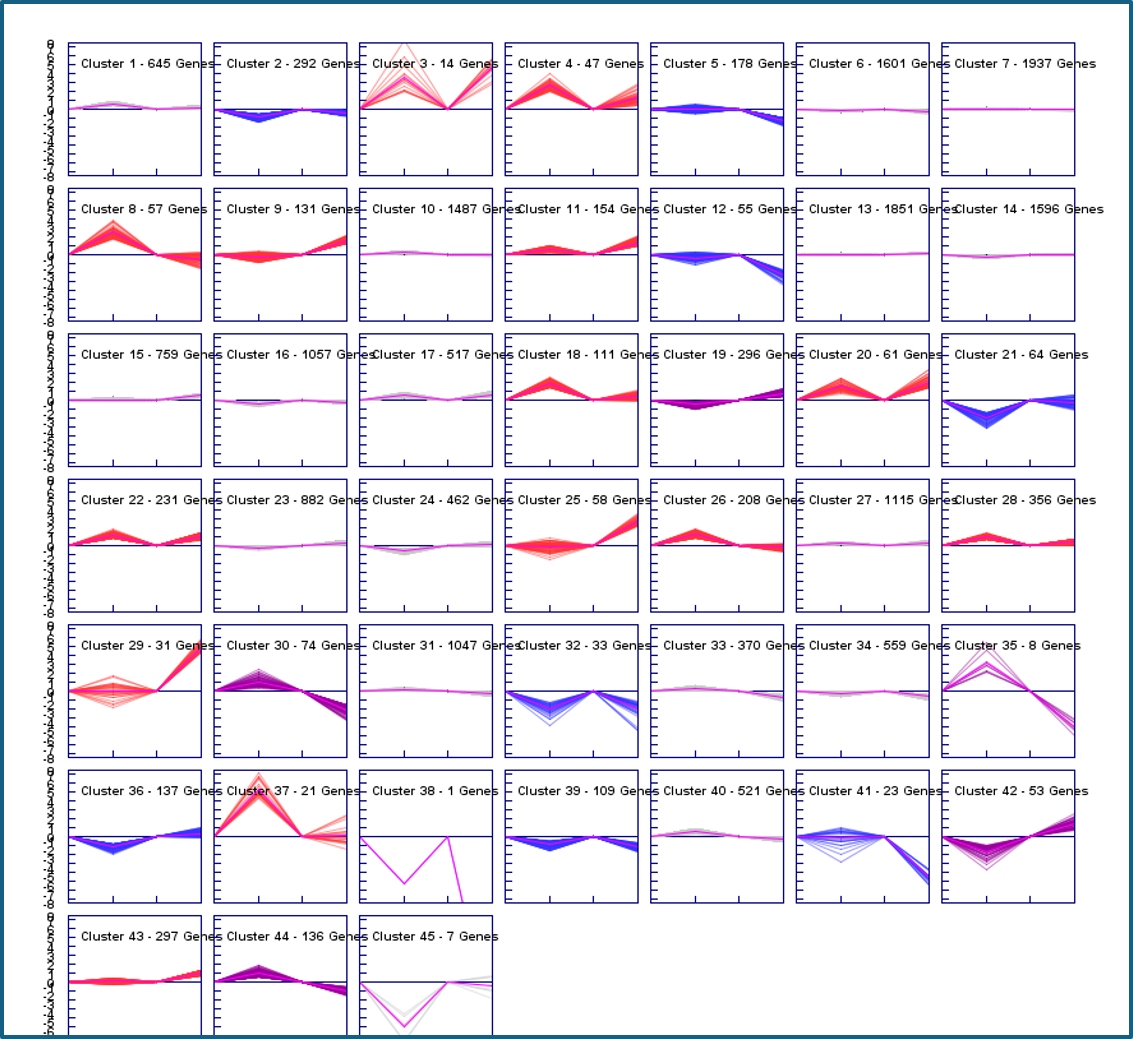

Supplement: Supplementary file 1 — Figure S1. Clusters in optimal conditions ‐ Clustering analysis conducted with Genesis, further normalization of log2FC. A partitional clustering analysis algorithm (k‐means) was used, which allows a set of objects to be divided into 45 groups on the basis of their trends on four comparison points: c5/c5, e5/c5, c24/c24 and e24/c24. The analyzed clusters in which the biostimulant induces a reduction of the expression at 5 or 24 hours are shown in blue, the clusters showing increases in the expression in red, the clusters containing genes with opposite trends between the 5 and the 24 hours sampling in purple. Lastly, in gray all clusters that were not selected for enrichment analysis. [file PPL-176-e70007-s004.tif]

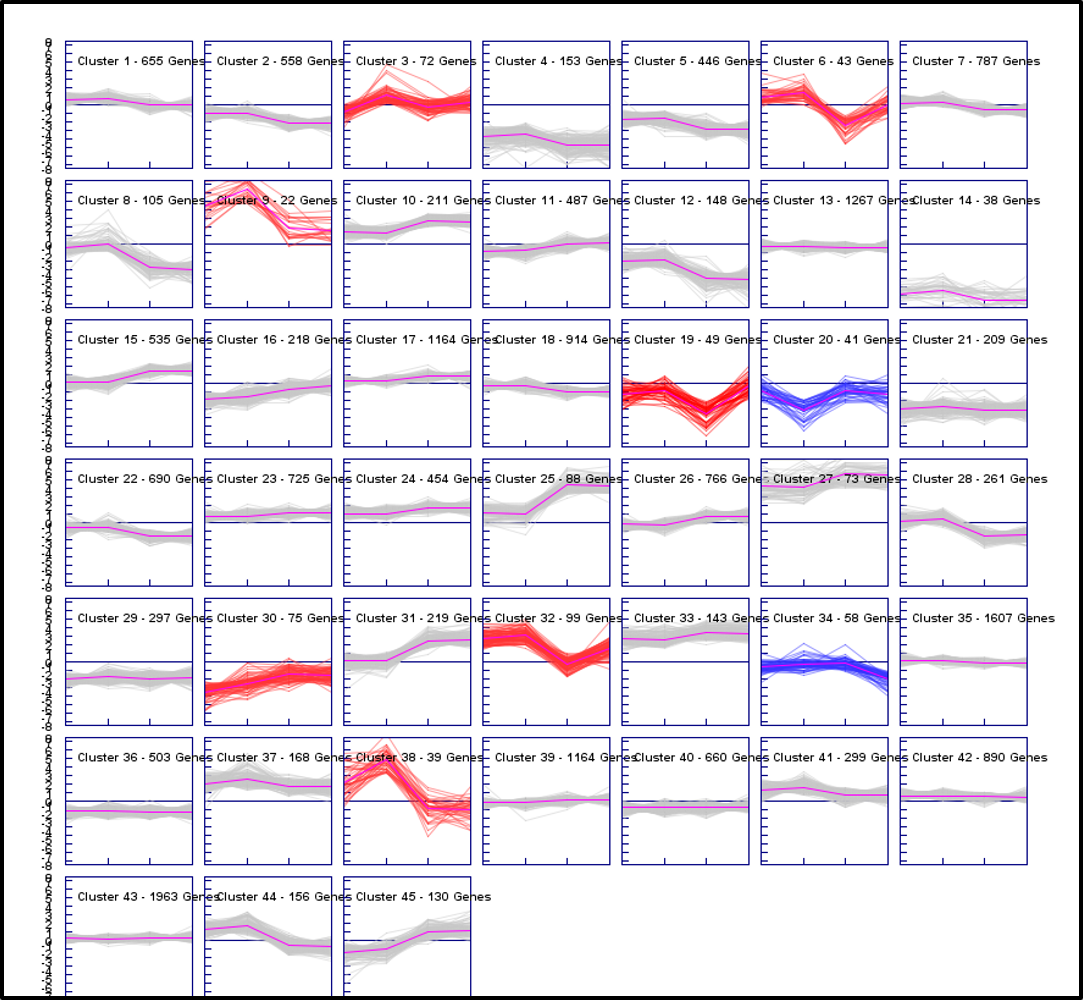

Supplement: Supplementary file 2 — Figure S2. Clusters in stressed conditions ‐ Clustering analysis conducted with Genesis, further normalization of log2FC. A partitional clustering analysis algorithm (k‐means) was used, which allows a set of objects to be divided into 45 groups on the basis of their trends on four comparison points: cs5/c5, es5/c5, cs24/c24 and es24/c24. The analyzed clusters in which the biostimulant induces a reduction of the expression at 5 or 24 hours are shown in blue, the clusters showing increases in the expression in red. Lastly, in gray all clusters that were not selected for enrichment analysis. [file PPL-176-e70007-s002.tif]
